# Persistence Length of Human Cardiac α-Tropomyosin Measured by Single Molecule Direct Probe Microscopy

**DOI:** 10.1371/journal.pone.0039676

**Published:** 2012-06-21

**Authors:** Campion K. P. Loong, Huan-Xiang Zhou, P. Bryant Chase

**Affiliations:** 1 Department of Biological Science, Florida State University, Tallahassee, Florida, United States of America; 2 Department of Physics, Florida State University, Tallahassee, Florida, United States of America; 3 Institute of Molecular Biophysics, Florida State University, Tallahassee, Florida, United States of America; German Cancer Research Center, Germany

## Abstract

α-Tropomyosin (αTm) is the predominant tropomyosin isoform in adult human heart and constitutes a major component in Ca^2+^-regulated systolic contraction of cardiac muscle. We present here the first direct probe images of WT human cardiac αTm by atomic force microscopy, and quantify its mechanical flexibility with three independent analysis methods. Single molecules of bacterially-expressed human cardiac αTm were imaged on poly-lysine coated mica and their contours were analyzed. Analysis of tangent-angle (θ(s)) correlation along molecular contours, second moment of tangent angles (<θ^2^(s)>), and end-to-end length (L_e-e_) distributions respectively yielded values of persistence length (L_p_) of 41–46 nm, 40–45 nm, and 42–52 nm, corresponding to 1–1.3 molecular contour lengths (L_c_). We also demonstrate that a sufficiently large population, with at least 100 molecules, is required for a reliable L_p_ measurement of αTm in single molecule studies. Our estimate that L_p_ for αTm is only slightly longer than L_c_ is consistent with a previous study showing there is little spread of cooperative activation into near-neighbor regulatory units of cardiac thin filaments. The L_p_ determined here for human cardiac αTm perhaps represents an evolutionarily tuned optimum between Ca^2+^ sensitivity and cooperativity in cardiac thin filaments and likely constitutes an essential parameter for normal function in the human heart.

## Introduction

Tropomyosin (Tm) is a dimeric, α-helical coiled-coil protein that binds actin and is found in a wide range of eukaryotic cells [1]. The α-tropomyosin (αTm) isoform is a splicing product of the human TPM3 gene transcript; it is commonly found in vertebrate striated muscle and is the predominant tropomyosin isoform in the normal heart of adult humans. The two polypeptide chains of an αTm molecule are aligned in parallel and in register [2], [3]. In striated muscle, Tm constitutes a crucial component of thin filaments for Ca^2+^-regulation of contraction. Systolic contraction is initiated by elevation of cytoplasmic Ca^2+^ that binds to the thin filament regulatory protein troponin (Tn), which undergoes a conformational change and induces azimuthal movement of Tm on the thin filament to uncover myosin binding sites. The primary structural regulatory unit responsible for this Ca^2+^ switch consists of one Tm molecule, one Tn complex, and seven actin monomers, although the functional regulatory unit may be larger as in skeletal muscle [4], or smaller as in cardiac muscle [5]. Currently available data can be most simply explained by a 3-state model for regulation of actomyosin interactions by Tn and Tm: a “blocked” state in the absence of Ca^2+^, a Ca^2+^-induced “closed” state, and a myosin-induced “open” state [6], [7], [8], [9]. The relationship between the mechanical properties of αTm and its regulatory function is widely speculated on but is not yet fully understood.

Modeling studies suggest that the presence of, and variations in, myofilament compliance could alter myocyte function at all levels of Ca^2+^-activation [10], [11], [12] and some aspects of fiber mechanics are most simply explained by Ca^2+^-dependent changes in sarcomere compliance [13]. Estimates of thin filament flexibility suggest that Tn-Tm modulate compliance in a Ca^2+^-dependent manner [14] and this could be directly influenced by flexibility of Tm. The flexibility of a linear, chain-like molecule such as Tm increases with temperature and can be described by its persistence length (L_p_), which is the length over which the chain loses directional correlation. L_p_ of α-helical coiled-coil regions of myosin and paramyosin at low temperature (7°C) was estimated by viscoelasticity measurements to be 130 nm [15]; a lower bound of L_p_ for the tail region of rabbit skeletal myosin was determined to be ∼100 nm when adsorbed to electron microscopy (EM) grids [16], albeit at unspecified temperature. Crystallographic and solution studies of WT rabbit cardiac αTm at 30°C yielded L_p_ estimates of 65 nm or 170 nm, depending on location in the crystal structure [17]. A more recent EM study measured the average L_p_ from 16 molecules of bovine cardiac αTm to be ∼102 nm, presumably at room temperature [18].

We present here the first direct images of WT recombinant human cardiac αTm proteins by atomic force microscopy (AFM), and corresponding L_p_ values at room temperature by three analysis methods involving tangent angle correlation along molecular contours, second moment analysis of tangent angles and distribution of the end-to-end lengths (L_e-e_) of molecules adhered on poly-lysine coated mica surfaces. These analyses yield values of L_p_ comparable to the contour length (L_c_), consistent across multiple samples independently prepared in identical conditions. Our results are consistent with previous reports that the size of a functional regulatory unit in cardiac muscle is similar to that of a structural regulatory unit, implying there is limited spread of cooperative activation via αTm into near-neighbor regulatory units in cardiac thin filaments [5]. We also note a general overestimation of L_p_ when fewer than ∼100 molecules are included in data analysis, which suggests a large data set is necessary for reliable estimates of L_p_ using similar techniques.

## Materials and Methods

### Protein Preparation

WT human cardiac αTm cDNA was previously cloned into a bacterial expression vector in our laboratory by Dr. Fang Wang. Bacterial expression and purification of the recombinant human cardiac αTm protein was accomplished as previously described [19], [20]. Regulatory function of the purified αTm has been established previously using motility assays [19], [20] and verified with a variety of functional assays reported in the literature [21], [22], [23], [24], [25]. To adjust surface density, protein samples were typically diluted to 1 nM with a buffer (2 mM MgCl_2_, 5 mM NaCl, and 20 mM TRIS-HCl pH 7.5) prior to deposition onto the imaging substrate.

### Surface Preparation for Poly-lysine Coated Mica

In preliminary experiments, αTm samples were imaged on freshly cleaved, untreated mica. Images from these preliminary samples did not contain structures consistent with single molecules of αTm. For all experiments reported here, αTm samples were imaged on poly-lysine (p-Lys) coated mica surfaces. Grade V-1 muscovite mica substrates (SPI Supplies, Westchester, PA) were mounted on glass microscope slides with epoxy. Slides were rinsed with water and ethanol, blown dry with compressed N_2_ gas and the mica freshly cleaved prior to p-Lys coating.

p-Lys coated mica was prepared by incubating freshly cleaved mica for 30 s, 2 min or 5 min with a 0.01% (w/v) p-Lys solution of 1000–5000 MW poly-L-lysine hydrobromide (Sigma-Aldrich, St. Louis, MO), rinsed with 600 µl dH_2_O and dried with compressed N_2_ gas. An αTm sample was then immediately deposited on the treated mica for subsequent imaging as detailed below.

### Atomic Force Microscopy (AFM)

200 µl of 1 nM αTm samples were deposited on p-Lys coated mica substrates and incubated for 30–900 s. The slides were subsequently rinsed with 600 µl dH_2_O dispensed with an electronic pipette at moderate speed, and blown dry with compressed N_2_ gas (regulated at 60–80 psi) aimed perpendicularly away from the mica surface.

**Figure 1:**
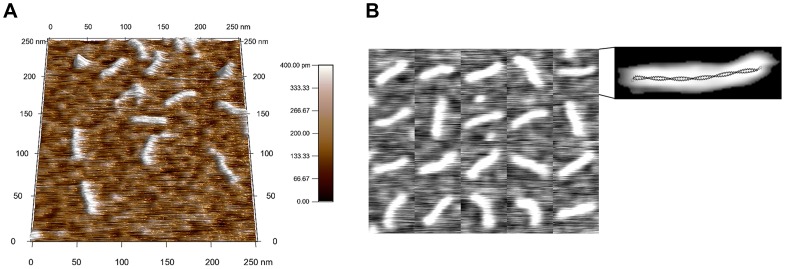
AFM images of α-tropomyosin (αTm) molecules. Wildtype human cardiac αTm was imaged dry on poly-lysine coated mica (A). Collage of 20 αTm molecules (B) show that molecular contours were smooth and continuous. One of the αTm molecules in the collage was processed as described (Figure 2 C) and overlayed with the x-ray structure of αTm [38] on the same scale (B, expanded on right), which is evidence that the AFM images were good representations of single αTm molecules.

AFM inspection was done in AC mode with an MFP-3D microscope (Asylum Research, Santa Barbara, CA) at room temperature. Olympus cantilevers with resonance frequency∼70 kHz were employed (Asylum Research, Santa Barbara, CA). The majority of images were acquired at 0.5 nm/pixel, while a subset of the data for a deposition time study (detailed below) was acquired at 1 nm/pixel. In both cases, tip convolution was the major limiting factor in image resolution. During each imaging session, the cantilever set-point was adjusted so that it barely tracked the surface topography in repulsive mode (i.e., phase angle <90 degrees); this minimized distortion and imaging artifacts caused by cantilever tip beating on the molecules. Resultant AFM images were processed in the MFP-3D software environment (Asylum Research, Santa Barbara, CA) by performing a 1^st^ order fit along each scan line followed by subtraction of a 1^st^ order plane from the whole image; all features with height above a program-determined threshold were masked out in the process, leaving images of the molecules unaltered while flattening the background (Fig. 1).

### Deposition Time: Variation of Numbers of Molecules on Substrate with Incubation Time

AFM images of αTm molecules deposited on p-Lys coated mica with different incubation times from 10–600 s were obtained as described above. For each incubation time, an area of 2.5×2.5 µm^2^ was surveyed at 1 nm/pixel. The number of αTm molecules within the area was counted manually. Only molecules that were more than half in the image frame were counted along the edges. Polymers of αTm formed by multiple single molecules were occasionally observed, in which case the equivalent number of single molecules was counted. Estimated uncertainties of 5% and 2 s were attributed to the counts and deposition time, respectively.

Ratios between the number of αTm molecules per cm^2^ adsorbed on the surface, *N_s_*, and the total number of molecules per cm^3^ of the 1 nM αTm sample, *N_B_(t = 0)*, were fitted against deposition time with a diffusion model [26]:
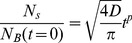
(1)where *D* is the diffusion constant of αTm. The non-linear least-square fitting was weighted by the inverse of the error in *N_s_/N_B_(t = 0)* and carried out in MATLAB (The MathWorks, Natick, MA).

### Image Processing and Skeletonization

A semi-automated image analysis GUI was developed in MATLAB using a modified version of the algorithm described by Brangwynne et al. [27]. All clearly separated and distinguishable elongated structures were processed using this algorithm for 199–1852 single molecules of WT αTm on multiple independently prepared samples. The algorithm consisted of three stages: 1) generation of 1-pixel wide skeleton from each individual αTm molecule; 2) refinement of skeleton to sub-pixel resolution by fitting the perpendicular height profile along the molecular contour; and 3) fitting the sub-pixel skeleton with a 5^th^ order polynomial to represent the continuous contour of the molecule.

**Figure 2:**
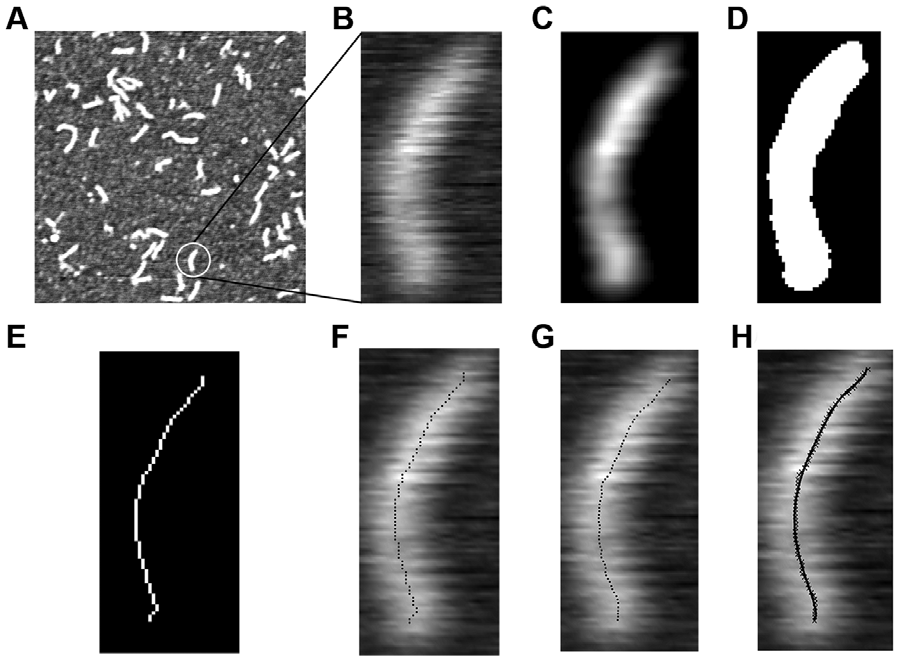
Image processing procedure to extract the molecular contour of αTm from a typical AFM scan. An αTm molecule was selected from a typical 512 nm ×512 nm scan (A) and cropped into a smaller image (B). The image was filtered by a Gaussian box-car filter (C), thresholded (D), and skeletonized into a 1-pixel wide connected contour (E, F). A refined skeleton with coordinates defined at sub-pixel precision was generated by fitting the perpendicular height profiles to a Gaussian function (G), which was then fitted with a 5^th^ order polynomial. The continuous contour defined by the polynomial conformed very well with the shape of the original molecule (H). Contour length (L_c_) and end-to-end length (L_e-e_) of the molecule shown were 41.7 nm and 38.4 nm, respectively.

In the first stage, AFM height measurements of αTm molecules on a p-Lys coated mica substrate were linearly mapped to a gray scale intensity image. The regions of the image containing individual αTm molecules were then cropped out (Fig. 2, A and B) and filtered by a local Gaussian kernel and an intensity averaging filter [28] (Fig. 2 C). Then a mask was generated such that all but the largest patch of connected pixels were eliminated within each region, leaving only the image of the molecule which was subsequently thresholded in MATLAB using Otsu’s method [29] (Fig. 2 D). The resulting binary image was skeletonized using a MATLAB routine by Howe [30] (Fig. 2 E). This yielded a single pixel-wide skeleton of connected pixels that represents a Tm molecule (Fig. 2 F). In the second stage, following Brangwynne et al. [27], the intensity profiles perpendicularly across the molecular contour at each skeleton point were fitted with Gaussian functions; the result was a new skeleton defined with sub-pixel resolution by the peak positions of the Gaussian fits. Skeletons with obvious artifacts such as acute kinks that deviated from the original image were discarded at this stage. Lastly, the new skeleton was fitted with a 5^th^ order polynomial to represent the Tm molecule’s contour. The skeletonization procedure likely missed small portions at the two ends of each molecule; to capture the entire contour length, we fitted the AFM height profiles beyond the ends of each skeleton with half of the 2D elliptical Gaussian function:
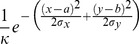
(2)where *κ* is the normalization factor, *a* and *b* are center coordinates, and σ_x_ and σ_y_ are the widths of the Gaussian along the x- and y-axes, respectively. The width of the Gaussian along the major axis (σ_x_) which was aligned with the contour at each end of the molecule was used to compensate for our calculation of contour length.

### L_p_ Determination by Tangent Angle Analyses

Angles, *θ*(*s*), between the tangents of the molecular contour at two points separated by segment length *s* were computed from the polynomial fit in 0.5 nm or 1 nm steps. The step size was chosen to coincide with the pixel resolution of our AFM images (i.e., maximum possible resolution achieved in the experiment) to avoid implicit interpolation beyond the limit of our technique. L_p_ is related to the average of cos*θ*(*s*) on *s* (<cos*θ*(*s*)>) by an exponential function [31], [32]:
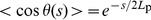
(3)where the factor of two in the exponent accounts for the fact that the molecular image was two-dimensional. The average, <cos*θ*(*s*)>, was taken both along the contour length of each skeleton and over different skeletons. L_p_ for WT αTm was obtained by linear regression on the logarithmically transformed data, weighted by *N_points_*×<cos*θ*(*s*)> where *N_points_* is the number of data points, and is reported with the fitting standard error.

A second estimate of L_p_ could be obtained from these data by analyzing the second moment of tangent angles (<θ^2^(s) >), as described by Frontali et al. [31], Rivetti et al. [26] and Mücke et al [33], [34]. In brief, the second moment of tangent angles from each sample was fitted against *s* according to the linear relation:
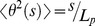
(4)where a zero intercept indicates the molecules were equilibrated on the substrate [31], [33], [34], and L_p_ was obtained from the slope of linear regression.

### L_p_ Determination by End-to-end Length Distribution

End-to-end length (L_e-e_) and contour length (L_c_) of each WT αTm molecule were calculated, respectively, as the linear distance between the two ends of the molecule and the integrated length along the polynomial fit. To accommodate the fact that the measured contour lengths varied from molecule to molecule, L_e-e_ of each molecule was scaled by its L_c_. The distributions of the scaled end-to-end lengths, l_e-e_, were fitted to that expected of a two-dimensional WLC [35]:
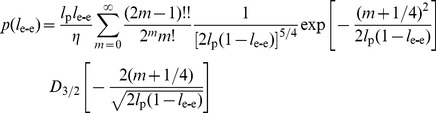
(5)where *l_p_  =  L_p_/L_c_*, *η* is a normalization factor, and *D_3/2_(x)* is a parabolic cylinder function. Eq. 5 is valid for chains with *l_p_*> ∼0.25. Least-squares fitting was carried out in MATLAB with a custom routine that utilized a MATLAB implementation of the parabolic cylinder function [36]; the sum of squared errors was minimized by changing the *l_p_* parameter with a step size of 0.0025. The errors on *l_p_* were estimated using the jackknife method [37].

### Estimation of L_p_ Dependence on Number of Molecules

The relationship of estimated L_p_ with number of αTm molecules analyzed, *N*, was investigated by random resampling of a large data set. 200 subsets of *N* (*N*  = 10, 20, 40…) single molecules of αTm were randomly selected from a total of 1852 single molecule images obtained from the same sample. Random selection was achieved by a “Mersenne Twister” pseudorandom number generator in MATLAB. For each value of *N*, 200 L_p_ estimates were computed with tangent angle correlation analysis from the corresponding subsets.

## Results

Figure 1 shows AFM scans of WT αTm on p-Lys (0.01% w/v) coated mica (Fig. 1 A). Elongated structures on p-Lys coated mica were clearly distinguishable and have lengths consistent with that expected for single αTm molecules (Fig. 1 B), as evidenced by an overlay of the αTm x-ray structure [38] (PDB ID: 2TMA) onto an AFM image of a WT αTm molecule (Fig. 1 B, expanded on right). In preliminary experiments, we also imaged αTm samples on freshly cleaved, untreated mica, but did not obtain structures consistent with single molecules of αTm (data not shown). Using highly sensitive assays of function–in vitro motility assays–we have previously demonstrated that positively charged surfaces are good substrates for functional binding of another molecule (myosin or the two-headed, proteolytic digest fragment of myosin called heavy meromyosin, HMM) that has an α-helical coiled-coil tail [39], [40], [41]. To achieve functional binding of myosin (or HMM), the primary mode of surface adsorption must be through the α-helical coiled-coil tail region rather than the motor domains, or heads [42]. Furthermore, we initially tested a wider range of p-Lys concentrations, but chose 0.01% (w/v) as the minimum concentration at which we observed statistically sufficient numbers of clearly distinguishable structures consistent with single molecules of αTm (data not shown).

**Figure 3:**
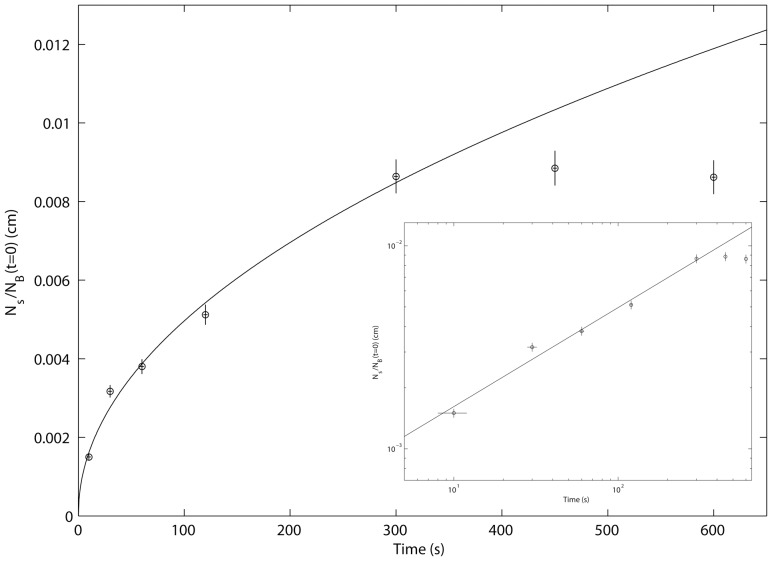
Deposition rate of αTm on p-Lys substrate shows the process is diffusion driven and irreversible. The number ratio between αTm molecules adhered to p-Lys coated mica and in 1 cm^3^ of the bulk solution, *N_s_/N_B_(t = 0)*, increased with incubation time up to 300 s, as shown in both linear (main graph) and logarithmic (inset) scales. 5% of total number of αTm molecules in the bulk solution were deposited on the substrate by the 300 s incubation; the absence of discernible change at longer incubation times of 450 s and 600 s suggests the top layer of the bulk solution was depleted of αTm molecules [26]. Fitting data from incubation time 10 s to 300 s to Eq. 1 (solid lines) returned estimates of two parameters: exponent parameter *p* equals 0.49, in close accordance to an irreversible and diffusion driven deposition process [26]; and diffusion constant parameter *D* of αTm equals 2.2×10^−7^ cm^2^/s, consistent with previous estimates [43].

The number of αTm molecules adhered to p-Lys coated mica increased with incubation time, up to 300 s, as shown in Fig. 3 in both linear (main graph) and logarithmic (inset) scales. No discernible change was observed for longer incubation times at 450 s and 600 s, suggesting the top layer of the bulk solution was depleted of αTm molecules [26]. Vertical and horizontal error bars equal to 5% of the corresponding *N_s_/N_B_(t = 0)* ratio and 2 s, respectively. Fitting data from incubation time 10 s to 300 s to Eq. 1 returned estimates of two parameters: exponent parameter *p* equals 0.49, in close accordance to an irreversible and diffusion driven deposition process [26]; and diffusion constant parameter *D* of αTm equals 2.2×10^-7^ cm^2^/s, consistent with previous estimates [43].

The measured L_c_ values of WT αTm on p-Lys coated mica surfaces were similar to that expected for single molecules of Tm (∼40 nm) [1], [44] (see also Fig. 1B, expanded on right) and variation is within 0.5–1 nm, or 1–2 image pixels (Table 1). The values of L_c_ reported include corrections of 4.1–4.8 nm to account for the chain length (σ_x_, Eq. 2) beyond the two end pixels. The mean L_e-e_ of all independently prepared samples of WT αTm on p-Lys coated mica are summarized in Table 1. Variation in L_e-e_ is within 0.7 nm, similar to that of L_c_. To test whether the variation in L_c_ contributed to the variation in L_e-e_, we normalized the L_e-e_ value of each αTm molecule by the L_c_ value of the same molecule. The values of mean l_e-e_ are again consistent across independently prepared samples. Measurements of L_c_ and l_e-e_ suggest our methodology is highly reliable and consistent across multiple samples prepared under identical conditions, as well as between different incubation times of p-Lys and WT αTm explored in this report.

**Table 1:** Summary of L_c_, L_e-e_, and L_p_ from tangent angle correlation, second moment and end-to-end length analyses.

Incubation time (seconds)	30*	300	600 #1	600 #2	600 #3	900**
Number of molecules	1852	199	741	628	798	979
Contour length, L_c_ (nm)	39.3±0.1	39.6±0.2	39.7±0.1	40.0±0.1	40.5±0.1	40.1±0.1
End-to-end Length, L_e-e_ (nm)	32.8±0.1	32.6±0.3	33.2±0.1	32.9±0.2	32.9±0.1	33.3±0.1
Normalized end-to-end length, l_e-e_	0.937±0.002	0.928±0.005	0.932±0.003	0.928±0.003	0.923±0.003	0.930±0.002
Persistence length, L_p_ (nm) by tangent angle correlation	48.3±0.7	42.3±0.4	45.8±0.8	43.5±0.8	40.6±0.8	45.5±0.7
Persistence length, L_p_ (nm) by l_e-e_ distribution	64.4±3.4	44.7±4.7	51.6±2.1	49.0±2.8	41.7±2.0	49.6±3.2
Persistence length, L_p_ (nm) by tangent angle second moment	45.9±0.3	40.9±0.2	44.5±0.4	41.9±0.3	39.5±0.3	44.0±0.3

* 0.01% p-Lys deposition on mica by 30 s incubation.

** 0.01% p-Lys deposition on mica by 300 s incubation.

**Figure 4:**
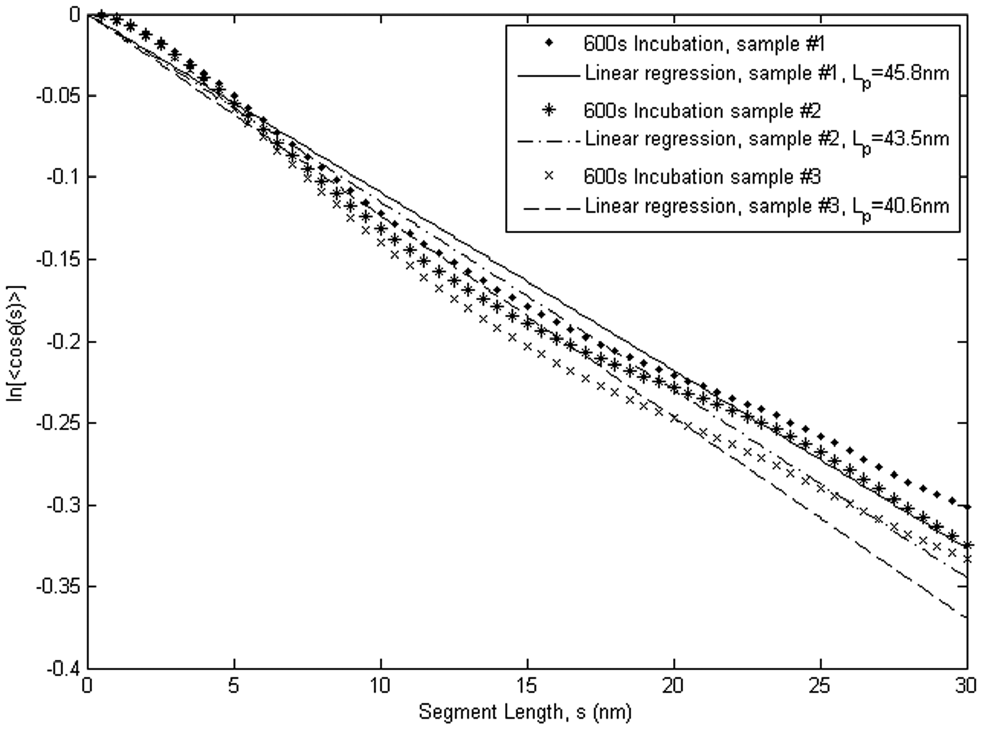
Tangent angle correlation analysis shows that L_p_ of WT human cardiac αTm equals 40.6−45.8 nm. ln(<cos(θ)>) data obtained from three separate samples independently prepared under identical conditions are plotted as a function of segment length along the molecular contour. The slope of this plot is –1/2L_p_. L_p_ for WT Tm from this analysis were 45.8±0.8 nm (N = 741, R^2^ = 0.99), 43.5±0.8 nm (N = 628, R^2^ = 0.98) and 40.6±0.8 nm (N = 798, R^2^ = 0.98). The variation in the L_p_ values represents the uncertainty inherent to our experimental setup and the tangent angle correlation analysis.

The results of tangent angle correlation analysis, which yielded L_p_ from ensemble averages of contour shape variation, are presented in Fig. 4 and Table 1. The L_p_ values of WT αTm measured from three independent samples prepared under identical conditions (2-min deposition of 0.01% p-Lys; 600 s deposition of 1 nM WT αTm) are 45.8±0.8 nm (N  = 742), 43.5±0.8 nm (N  = 628) and 40.6±0.8 nm (N  = 798). The ∼5 nm variation in these measurements represents the variability inherent to our methodology and experimental setup. As noted above, L_p_ is the length along the molecular contour over which the tangent vectors of the chain lose correlation. For a chain with a given contour length that is fixed at one end, the region that is explored by the other end increases when L_p_ becomes smaller. The results in Fig. 4 imply that the correlation length of WT αTm, or the limiting length along which mechanical signal can effectively propagate, is comparable to the contour length L_c_ of the molecule.

To test if tangent angles satisfy Gaussian statistics, and thus whether αTm molecules have equilibrated on the p-Lys coated substrate, we further analyzed the tangent angle data according to Frontali et al. [31] and Mücke et al. [33], [34]. First, we fitted the distribution of tangent angles at various segment lengths (*s*) to Gaussian functions. For segment lengths of 10 nm, 20 nm or 30 nm, the data were consistent with Gaussian functions (R^2^>0.98). Second, linear least squares regression of the second moment of tangent angles (<θ^2^(s)>) vs segment length using Eq. 4 passes through the origin and, from the slope (1/L_p_), yielded persistent length estimates (Table 1) that are consistent with tangent angle correlation analysis. Third, the ratio of the fourth moment (<θ^4^>) and <θ^2^>^2^ was close to 3 for *s* >10 nm. Taken together, these results indicate that αTm molecules were equilibrated on the substrate, and thus our estimates of L_p_ from the tangent angle data legitimately reflect mechanical flexibility of the protein [31], [33], [34].

**Figure 5:**
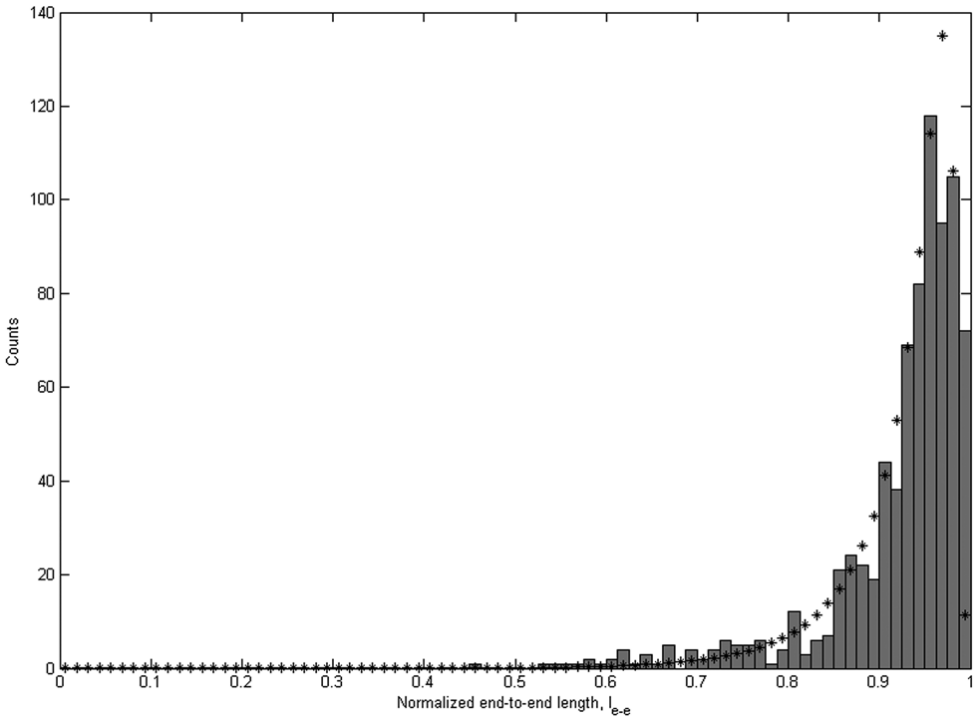
End-to-end length analysis shows L_p_ of WT human cardiac αTm consistent with tangent angle correlation analysis. Normalized end-to-end length (l_e-e_) distributions from one of the WT αTm samples incubated on p-Lys coated mica substrate for 600 s (N = 798) fits to the WLC model (Eq. 3). *l_p_* value from the fit was 1.0425±0.0505 (R^2^ = 0.88). Errors were estimated by the jackknife method (Materials and Methods).

To verify the L_p_ values obtained from tangent angles, we fitted the distributions of l_e-e_ with the WLC model (Eq. 5). Figure 5 shows the l_e-e_ distribution and the corresponding WLC fit for one sample of WT αTm deposited on p-Lys coated mica with 600 s incubation. This analysis yielded *l_p_*  = 1.04±0.05. Assuming 40 nm for the contour length, this is equivalent to a persistence length of 41.7±2.0 nm, in close agreement with the result from tangent angle correlation analysis for the same sample (L_p_ equals 40.6±0.8 nm). The values of L_p_ estimated by tangent angle correlation, second moment and end-to-end length for different samples and experimental conditions are summarized in Table 1. L_p_ obtained by end-to-end length analysis is generally longer than that by the tangent angle correlation analysis, but both are in the order of 1 to 1.3 contour lengths of WT αTm. The results from these analyses are consistent over different deposition times, as well as multiple samples independently prepared under identical conditions.

**Figure 6:**
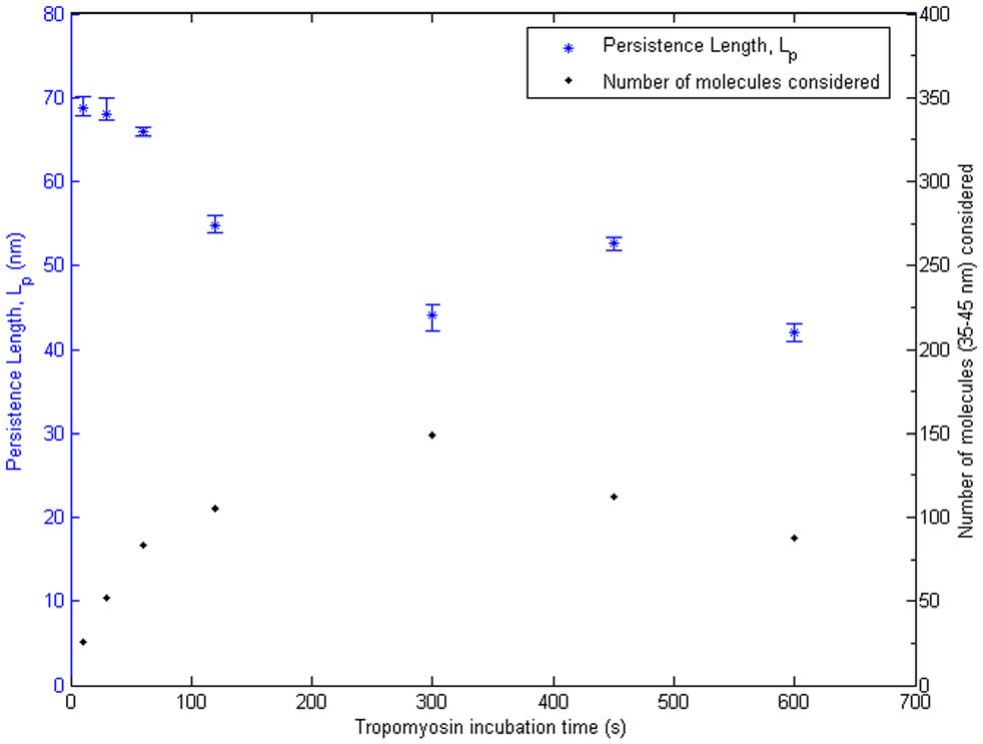
Deposition time study suggests L_p_ measurements were stable with incubation time of 300 s or longer. L_p_ values obtained by tangent angle correlation analysis (blue asterisks, left axis) and the corresponding number of molecules considered (black dots, right axis) were plotted against incubation times. An overestimation of L_p_ was observed at incubation times below 300 s, which may be due to the shorter incubation time and/or smaller number of molecules available for the analysis. L_p_ measurements were stable at incubation times above 300 s, where variation was comparable to the inherent uncertainty in our methodology. This implies an incubation time of 300 s was sufficient for surface equilibration of αTm on p-Lys coated mica substrate.

To further understand the effect of deposition time on the values of L_p_ measured with this methodology, we analyzed the 5×5 µm^2^ survey images previously obtained at 1 nm/pixel. Figure 6 shows the variation of L_p_ estimates, obtained from tangent angle correlation analysis, with deposition times from 10 s to 600 s. Number of αTm molecules available for analysis in the surveyed area was significantly smaller at short deposition times, with only 26 molecules at the 10 s time point. Estimated values of L_p_ gradually decreased and stabilized with increasing deposition time. It is unclear whether the longer L_p_ estimates were due to short incubation times or the relatively smaller number of molecules. Nonetheless, this result suggests a deposition time of at least 300 s is sufficient for surface equilibration of αTm and a reliable measurement of its L_p_.

**Figure 7:**
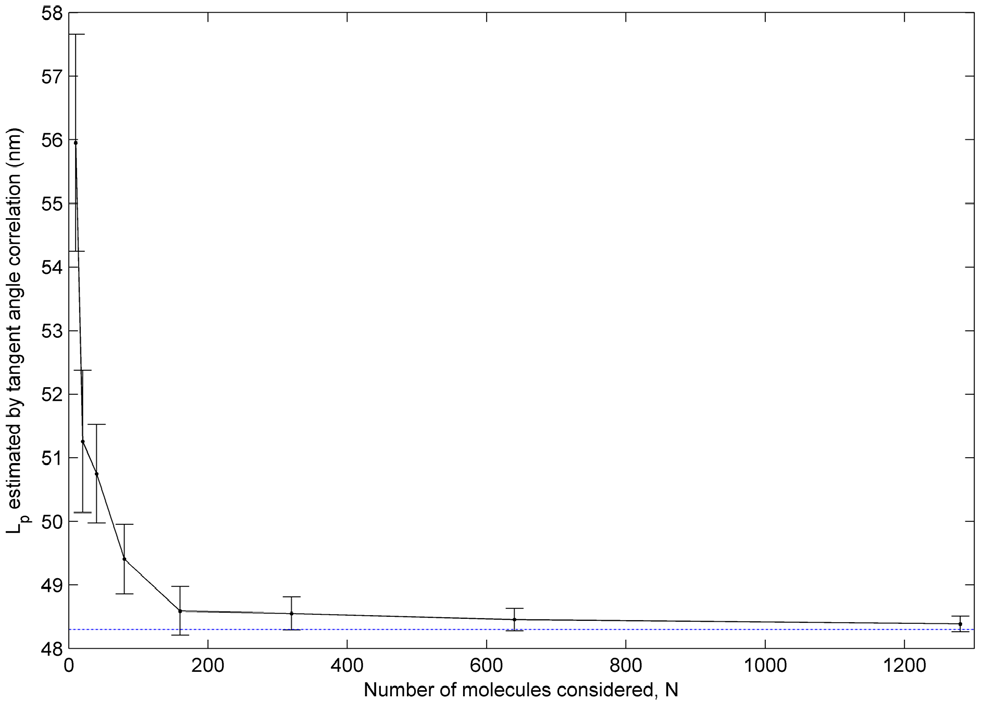
A sufficiently large population of αTm molecules is required for reliable measurement of L_p_. L_p_ was calculated for each of 200 subsets of N αTm (N = 10, 20, 40…) randomly selected from a total of 1852 molecules. The mean L_p_ is plotted against N. A consistent overestimation of L_p_ up to 8 nm was observed for small N (<80–160). This overestimation can be attributed to the biased distribution of contour shapes, where highly bent configurations of the molecule are relatively rare. In case of small N, therefore, the highly bent configurations are under- or un-sampled, which leads to overestimation of L_p_ based on a biased population of relatively straight molecules.

Figure 7 shows the dependence of measured L_p_ on number of molecules considered in the tangent angle analysis. Values of L_p_ are computed from subsets of 10 to 1280 αTm molecules randomly selected from a total of 1852 molecules on a single sample. We observe a significant overestimation of L_p_ by up to ∼8 nm when fewer than ∼80–160 molecules were considered in the analysis. Similar trends are observed in the values of L_p_ obtained using second-moment analysis of tangent angles (data not shown) and from various sized samples out of 5000 simulated worm-like chains with L_c_  = 40 nm and L_p_  = 44 nm (Fig. S1). This result suggests the small sample sizes may explain the overestimation of L_p_ at short incubation times in Fig. 6, and that a minimum population of about 80–160 molecules is needed for a reliable estimate of αTm L_p_.

## Discussion

In this study, we obtained AFM images of individual WT human cardiac αTm molecules and measured the persistence length. Correlation and second moment analyses of tangent angles resulted in L_p_ values of 40.6–45.8 nm and 39.5–44.5 nm, respectively. Fitting of the end-to-end length distribution to the WLC model resulted in similar L_p_ values of 41.7–51.6 nm. Measurements of L_p_ are consistent over multiple samples with incubation times of 300 s or longer. A sufficiently large sample size–with 100 molecules or more–is required for a reliable estimate of L_p_ (Fig. 7). Our measurements are consistent across multiple, independently prepared samples (Table 1), and suggest that the L_p_ of WT human cardiac αTm is equivalent to 1–1.3 molecular contour lengths (L_c_).

We were able to obtain AFM images of clearly separated and distinguishable, elongated structures on p-Lys coated mica surfaces (Fig. 1), and the lengths of these structures were consistent with that expected for single αTm molecules. While single-molecule images of rotary-shadowed bovine cardiac and chicken gizzard smooth muscle αTm have been previously obtained by electron microscopy [18], [45], the data from this study are to our knowledge the first direct, AFM images of surface-adhered, single human cardiac αTm molecules. Resolution of the images was mostly limited by convolution with cantilever tips. The deposition rate of αTm onto p-Lys coated mica substrate resulted in an estimated diffusion constant consistent with that obtained by other methods [43], and suggests the deposition process is both diffusion driven and irreversible [26], [46] (Fig. 3). Using the semi-automated image processing routine described in Materials and Methods (Fig. 2), the shapes of more than 5000 single WT αTm molecules in total from multiple, independently prepared samples were analyzed.

We quantified the flexibilities of the protein molecules by three independent methods involving tangent angle correlation (Eq. 3; Fig. 4), tangent angle second moment (Eq. 4), and end-to-end length distribution (Eq. 5; Fig. 5). Tangent angle correlation analysis is often applied to images of molecules and polymers obtained from microscopy [14], [18], [47]. Accurate measurement of L_p_ using surface adsorption techniques such as in the present study requires full equilibration of the molecules. We showed that our data fulfill this condition by verifying the Gaussian distribution of tangent angles for various segment lengths, as suggested by Frontali et al. [31]. It is assumed that the washing and drying procedures do not introduce artifacts that affect our L_p_ measurements. This assumption is partly justified by the high level of consistency between L_p_ values obtained between multiple samples independently prepared under either identical or a range of conditions. In principle, this concern can be alleviated by imaging the molecules in solution, but it is often not possible to obtain similarly high resolution images in solution. Conventionally, second moment analysis is also utilized to demonstrate equilibration of filamentous molecules imaged with scanning probe microscopy on 2D substrates [26], [31], [33], [34]. L_p_ has previously been estimated in a variety of molecules other than Tm such as F-actin [14], [27], [47], [48], microtubules [27], [48], DNA [26], [31], [49], RNA [50] and trimeric type-I tropocollagen [51] using data obtained by observation of thermal fluctuations, force-extension relations [49], [50], [51], [52] or end-to-end lengths [50]. Many of these approaches rely on the WLC model to obtain L_p_. Here we utilized independent analyses of our data to show that fitting the end-to-end length distribution to the WLC model provides an important check on the reliability of L_p_ values from tangent angle analyses.

We estimated the inherent variability in our methodology of measuring L_p_ to be ∼5 nm by tangent angle correlation and second moment analyses, and ∼10 nm by the WLC analysis, from three separate samples of surface adhered αTm independently prepared in identical conditions (Fig. 4 and Table 1). We showed, by analysis of <θ^2^(s)>, that αTm molecules were equilibrated on the substrate (Results). In addition, L_p_ varied little when αTm was incubated for at least 300 s (Table 1). The values of L_p_ obtained from tangent angle correlation, second moment <θ^2^(s)>, and end-to-end length analyses are consistent with each other (Table 1), which bolsters confidence in the reliability of our result. The αTm molecule has an intrinsic curvature which allows for binding to the helical structure of F-actin [38], [53], [54]. It has been argued, in the case of curved DNA molecules, that the intrinsic or static curvature of a molecule should be distinguished from the dynamic changes in contour shapes due to its mechanical flexibility [55]. A reciprocal relation was proposed to decouple the corresponding persistence lengths due to intrinsic curvature (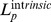) and mechanical flexibility (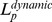) from the experimentally measured value (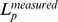):
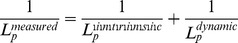
(6)For a given intrinsic curvature, the effect of 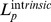 in Eq. 6 diminishes as mechanical flexibility increases [56]. In our case, assuming a similar intrinsic curvature previously estimated for bovine cardiac αTm [18], the resultant 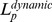 is about 67 nm, or 1.7 L_c_. Therefore, Eq. 6 does not produce a pronounced correction to the experimentally measured L_p_ of human cardiac αTm.

We statistically demonstrated that a sufficiently large number of molecules is necessary to avoid overestimating the L_p_ of αTm (Fig. 7 and Fig. S1). This can be best explained by the biased distribution of contour shapes for semi-flexible and rigid molecules, such as αTm. According to the WLC model, it is relatively unlikely for semi-flexible or rigid molecules to assume highly bent configurations. This bias is evident in the end-to-end length distribution (Fig. 5 and Fig. S2), where bins corresponding to relatively straight molecules are much more populated than that of highly bent configurations. When a measurement of L_p_ is obtained from a small number of randomly selected molecules, it is likely that most of the molecules are relatively straight, leaving the more bent configurations under- or un-sampled (Fig. S2). The result is an overestimation of L_p_ based on a limited selection of mostly straight molecules. Therefore, a sufficiently large selection of molecules is necessary to sample all possible configurations of contour shapes, and to obtain a reliable measurement of L_p_. Our measurements of αTm L_p_ are to our knowledge the first to be based on large numbers of molecules, with at least ∼200 single αTm molecules (typically >500) from each experimental condition.

Our result of L_p_ for WT human cardiac αTm is near the low end of the range of previous L_p_ measurements of rabbit and bovine cardiac αTm [17], [18]. This might be attributed to difference in experimental techniques, temperature when the samples were prepared and in which the measurements were obtained, and sample sizes of single molecule studies as reasoned above. However, it is also possible that different Tm isoforms within an organism, or the proteins from different organisms could have different flexibilities (see next paragraph). The mechanical properties of αTm are likely to play a central role in its Ca^2+^-regulatory function [56]. Therefore, although beyond the scope of this study, it can be speculated that Tm’s in different organisms have evolved to have flexibilities suited to their cellular conditions.

During thin filament activation, αTm is mechanically displaced following Ca^2+^-binding to the Tn complex to expose myosin-binding sites of the underlying actin filament. Since the flexibility of αTm governs the transmission of this cooperative activation signal, our result implies that cooperative activation of human cardiac thin filaments spans approximately one structural regulatory unit, or 7 actin monomers. This is in line with a previous study [5] which showed that the functional regulatory unit in cardiac muscle is less than 7 actin monomers, while that in skeletal muscle is 12–14 actin monomers [4]. In addition, the mechanical barrier to initiate αTm movement on the thin filament is also governed by the flexibility of the αTm molecule. Our result suggests that this barrier is comparable to the torque required to displace an entire αTm molecule and its connected neighbor on the thin filament. Therefore, the flexibility of αTm modulates both the cooperativity in transmission of activation along and the Ca^2+^-sensitivity of activation of the thin filament. These factors are especially important during systole, which in cardiac muscle involves only subsaturating level of Ca^2+^: if L_p_<<L_c_, neighboring segments of αTm are essentially independent and thin filament activation will be less coordinated; conversely, if L_p_>>L_c_, the high mechanical barrier to initiate αTm movement implies that thin filament activation during systole will become highly unlikely [56]. We therefore suggest that our measured L_p_ of human cardiac αTm represents an evolutionarily tuned optimum between sensitivity and cooperativity in the system of Ca^2+^-regulated thin filament activation in the human heart.

### Conclusion

Recombinant WT human cardiac αTm molecules were imaged by AFM. Nearly identical L_p_ of about 41–52 nm were obtained under various experimental conditions using tangent angle correlation and second moment analyses, and fitting the end-to-end length distribution to the WLC model. Random resampling suggests a sufficiently large population, with at least 100 αTm molecules, is required for reliable estimates of L_p_. Our result that L_p_ of αTm equals ∼1–1.3 L_c_ is consistent with a previous study showing that the functional regulatory unit is approximately the same size as the structural regulatory unit in cardiac muscle, provided Tm flexibility is a major determinant of the spread of cooperative activation along thin filaments. We propose that the measured L_p_ represents an optimal flexibility of human cardiac αTm. As a result of this optimal flexibility, it is further speculated that a balance between Ca^2+^ sensitivity and cooperativity in cardiac thin filaments is achieved and likely constitutes an essential parameter for normal function in the adult human heart.

## Supporting Information

Figure S1 ***L*** **_p_ estimated from various sized samples of worm-like chains, showing overestimation when only a small number of chains are analyzed.** 5000 3-dimensional worm-like chains with L_c_  = 40 nm and L_p_  = 44 nm were generated. Tangent angle correlation analysis of the whole population yielded L_p_  = 44.7 nm (blue dashed line). From the whole population, a sample of N chains (N = 10, 20, 40 …) was randomly selected 200 times. *L*_p_ was then estimated from tangent angle correlation for each sample; mean and standard error of the mean were finally calculated from the 200 repetitions. A consistent trend of overestimated *L*_p_, similar to that in the analysis of αTm molecules (Figure 7), was observed for small N (i.e., <80–160). The standard error of the mean, shown as vertical error bars, shows that the systematic overestimation is not due to the inherent variability of L_p_ at small N.
(TIF)

Figure S2 **Asymmetric distribution of end-to-end lengths leading to under-sampling of highly bent configurations in small population of αTm molecules.** The normalized end-to-end length (l_e-e_) distributions of various sized samples (black) from a total of 1852 αTm molecules are overlaid on the distribution of all the molecules (grey). The number (N) of molecules in each sample increases from 10 to 400 (top to bottom; left to right). The same information is shown in expanded scales in the insets to highlight distributions of the subsets. For N <100, most molecules in the samples were relatively straight, as evidenced by the long normalized l_e-e_ (typically >0.85); more bent configurations, or molecules with short l_e-e_, were under- or un-sampled. In contrast, l_e-e_ distributions of samples with larger N (>100) contain more bent configurations and better resemble the l_e-e_ distribution of all the molecules. Therefore, L_p_ will be overestimated by analyses based on small numbers of molecules, as the molecules with more bent configurations are under-counted.
(TIF)
