# Supplementary figures and images for: Persistence Length of Human Cardiac α-Tropomyosin Measured by Single Molecule Direct Probe Microscopy
Source: PLoS One. 2012 Jun 21;7(6):e39676. doi: 10.1371/journal.pone.0039676 (PMC3380901; doi:10.1371/journal.pone.0039676)

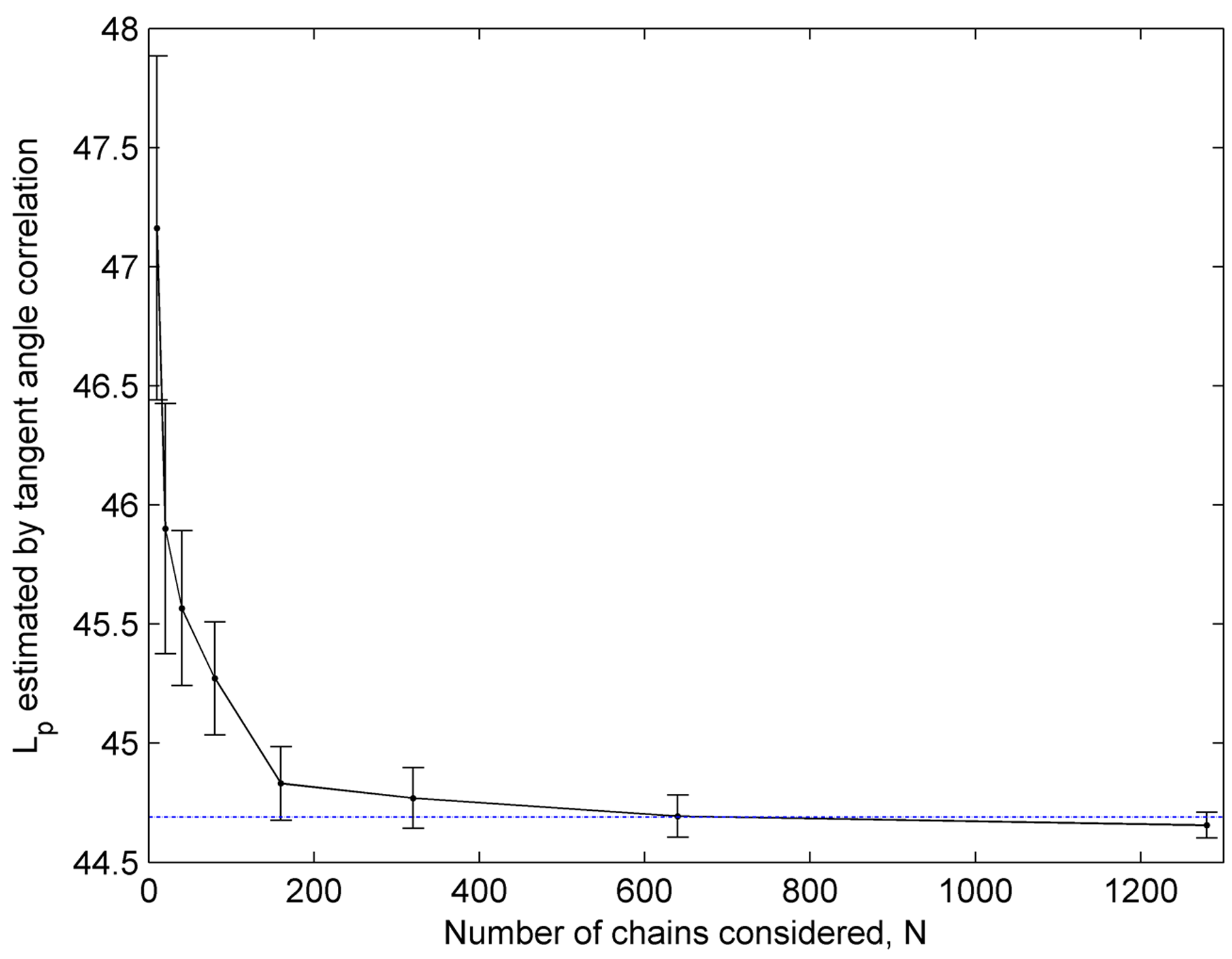

Supplement: Figure S1 — L p estimated from various sized samples of worm-like chains, showing overestimation when only a small number of chains are analyzed. 5000 3-dimensional worm-like chains with Lc = 40 nm and Lp = 44 nm were generated. Tangent angle correlation analysis of the whole population yielded Lp = 44.7 nm (blue dashed line). From the whole population, a sample of N chains (N = 10, 20, 40 …) was randomly selected 200 times. L p was then estimated from tangent angle correlation for each sample; mean and standard error of the mean were finally calculated from the 200 repetitions. A consistent trend of overestimated L p, similar to that in the analysis of αTm molecules (Figure 7), was observed for small N (i.e., <80–160). The standard error of the mean, shown as vertical error bars, shows that the systematic overestimation is not due to the inherent variability of Lp at small N. (TIF) [file pone.0039676.s001.tif]

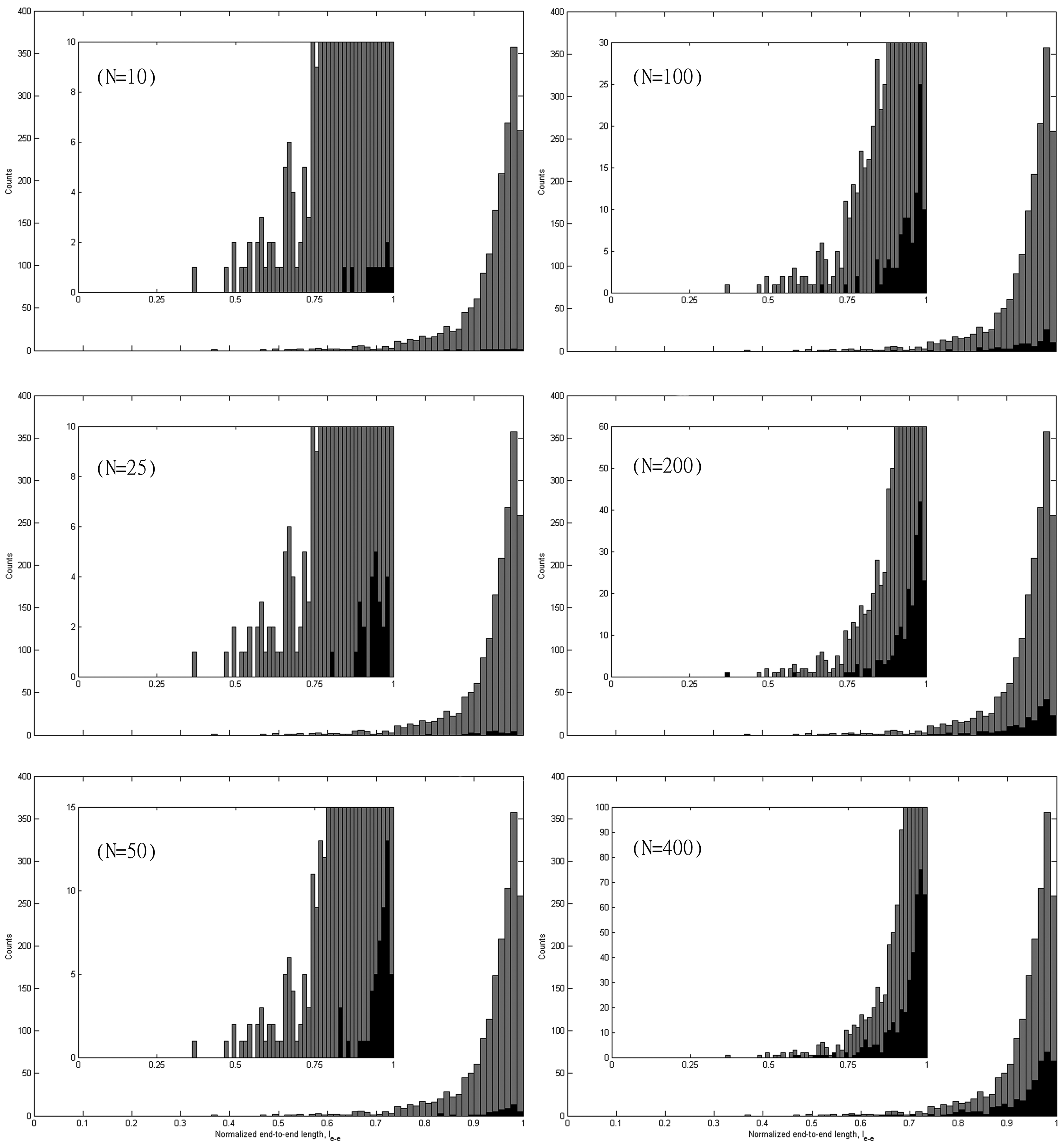

Supplement: Figure S2 — Asymmetric distribution of end-to-end lengths leading to under-sampling of highly bent configurations in small population of αTm molecules. The normalized end-to-end length (le-e) distributions of various sized samples (black) from a total of 1852 αTm molecules are overlaid on the distribution of all the molecules (grey). The number (N) of molecules in each sample increases from 10 to 400 (top to bottom; left to right). The same information is shown in expanded scales in the insets to highlight distributions of the subsets. For N <100, most molecules in the samples were relatively straight, as evidenced by the long normalized le-e (typically >0.85); more bent configurations, or molecules with short le-e, were under- or un-sampled. In contrast, le-e distributions of samples with larger N (>100) contain more bent configurations and better resemble the le-e distribution of all the molecules. Therefore, Lp will be overestimated by analyses based on small numbers of molecules, as the molecules with more bent configurations are under-counted. (TIF) [file pone.0039676.s002.tif]
